# Study on Underground Sewage Pipeline Temperature Based on OFDR Technology and Numerical Simulation Methods

**DOI:** 10.3390/s26041316

**Published:** 2026-02-18

**Authors:** Lei Gao, Xinyu Wu, Zhuodi Zheng, Mengran Guo

**Affiliations:** Key Laboratory of Ministry of Education for Geomechanics and Embankment Engineering, Hohai University, Nanjing 210024, China; wuxinyu94@hhu.edu.cn (X.W.); zhuodi_zheng@163.com (Z.Z.); mrguo@hhu.edu.cn (M.G.)

**Keywords:** field test, numerical simulation, optical frequency domain reflectometry, sewage pipeline, temperature

## Abstract

The underground sewage pipeline is one of the lifeline projects of the city. The pipeline temperature is one of the important influencing factors for the safe operation of the underground sewage pipeline. This study is based on the sewage pipeline project on Jianning Road in Nanjing; the sewage pipeline temperature monitoring experiment was conducted first. The optical frequency domain reflectometer (OFDR) technology was used to monitor the sewage pipeline temperature. The numerical simulation method was also incorporated to study the variations in sewage pipeline temperature. The optical fiber monitoring data for the underground sewage pipeline temperature were collected, and the spatiotemporal distribution of the underground sewage pipeline temperature was explored. The results show that the underground sewage pipeline temperature is continuously rising, and the rate of increase is slow. The maximum temperature change is 0.55 °C. The numerical simulation results are consistent with the trend of the measured results. The findings will provide a valuable reference for further research on sewage pipeline temperature.

## 1. Introduction

The urban underground sewage pipelines are one of the lifeline projects of cities, as they undertake the function of transporting urban domestic and production wastewater. They are also one of the key infrastructures for improving the internal water environment quality of cities and preventing urban rainfall and waterlogging. During the operation of sewage pipelines, damage accidents often occur; pipeline deformation is one of the main causes of pipeline damage [1]. The temperature can affect the structural durability of pipelines; the impact of pipeline temperature changes on pipeline structural damage cannot be ignored [2]. The commonly used sewage pipeline detection technologies currently include television detection technology, laser detection technology, periscope detection technology, infrared thermal imaging detection technology, and ultrasonic detection technology [3]. When these traditional detection technologies are compared, there are shortcomings. For example, periscope detection technology not only has high energy consumption but also needs to enter the pipeline, which is difficult to maintain in large-scale, long-term monitoring [4]. At the same time, the complex closed environment of underground sewage pipes (sewage and silt) may cause damage to the delicate and fragile monitoring instruments. Long-term monitoring is the biggest test of the waterproofness and durability of sensors [5].

The distributed optical fiber sensing technology can obtain the changes in optical signals along the optical fiber path; the physical parameters, such as temperature and strain, can be accurately measured. The distributed optical fiber sensing technology can be used in the field of geotechnical engineering monitoring [6,7,8,9]. This technology has advantages, such as superior waterproofing, good durability, and long-distance sensing [10]. In recent years, the optical frequency domain reflection fiber sensing technology (OFDR) has gradually gained attention as a new distributed fiber monitoring technology [11]. In research by Gao, L., results indicated that OFDR technology has advantages in providing high spatial resolution and high-precision measurement of geotechnical engineering [12,13,14,15,16,17]. More scholars use OFDR technology to monitor the temperature changes in various pipelines [18,19]. Boldyreva et al. [20] used OFDR technology to monitor temperature changes in nuclear fast reactor pipelines, and the liquid sodium leaks on the pipeline surface were detected and located accurately and safely. Brister et al. [21] used OFDR technology to monitor the temperature of natural gas pipelines to determine the structural health status of the pipelines. Li H.J. et al. [22] combined ADTS-OFDR technology and used it to monitor the process of pipeline leakage. The study showed that the location and leakage rate of pipeline leakage can be estimated by monitoring the maximum soil temperature rise, achieving early detection of pipeline leakage.

In summary, the existing research on pipeline temperature monitoring mostly focuses on oil, gas, and water supply pipelines; there have been no relevant reports on the temperature changes in urban underground sewage pipelines. Based on the underground sewage pipeline construction project on Jianning Road in Nanjing, this study carried out the temperature monitoring experiment on the sewage pipeline for the first time. The OFDR distributed optical fiber sensing technology was used to monitor the temperature of the sewage pipeline. The temperature change data of the pipeline was obtained. The numerical analysis model of the sewage pipeline was established. The spatiotemporal variation law of pipeline temperature was analyzed. The study provides scientific reference for the research on urban sewage pipeline temperature.

## 2. Materials and Methods

### 2.1. Optical Fiber Monitoring System for Sewage Pipeline Temperature

**Figure 1:**
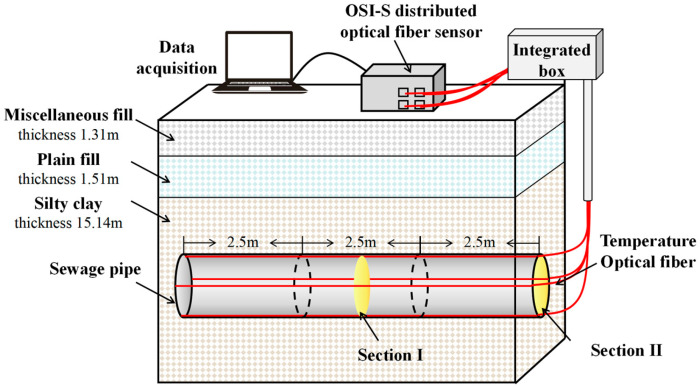
High-precision optical fiber monitoring system of underground sewage pipeline temperature here.

**Figure 2:**
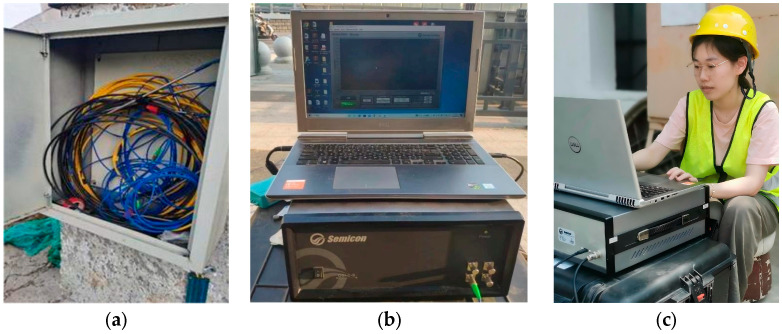
On-site monitoring: (**a**) integrated box; (**b**) monitoring equipment; and (**c**) field monitoring.

In order to meet the demand for sewage transportation and discharge in Nanjing, the complete sewage pipeline network was built, and the Nanjing Municipal Government has launched the Jianning Road Sewage Pipeline Project. The project is located on the section of Jianning Road, Central North Road to Siping Road in Gulou District, Nanjing; it has a total length of 3 km and a pipeline diameter of 1.8 m. This study selects the sewage pipeline with a length of 50 m as the research object, which is composed of 20 section pipes; the length of each pipe is 2.5 m. The construction method of pipe jacking is used for the sewage pipeline; the grade III precast reinforced concrete pipe is selected. In order to accurately grasp the temperature changes in the sewage pipeline, this study developed a high-precision optical fiber monitoring system for underground sewage pipeline temperature. It includes the underground sewage pipeline, OFDR monitoring equipment, optical fiber sensor, integrator, optical fiber jumper, etc. The OFDR monitoring equipment used in the experiment is the OSI-S equipment produced by Hong Kong Donglong Technology Company. The equipment can achieve 1 mm spatial resolution and ±1.0 μ ε sensing accuracy within a monitoring range of 100 m. In this experiment, four optical fiber temperature sensors were used to monitor the temperature of the pipeline. The optical fiber sensors are made of plastic-encapsulated armored temperature optical cables, which are produced by Suzhou Nanzhi Sensing Technology Co., Ltd. (Suzhou, China), model NZS-DTS-C05. The optical fiber sensors are installed on both sides, the bottom, and the top of the sewage pipeline. The optical cable is laid on the inner wall of the pipeline through the steps of line fixing, grinding, dust removal, welding, and sticking, and the whole line is sealed with a stainless-steel arc cover plate to avoid the influence of the wastewater or debris on the monitoring results. The high-precision optical fiber monitoring system for underground sewage pipeline temperature is shown in Figure 1. The on-site monitoring situation is shown in Figure 2, and the spatial resolution of the data acquisition equipment is set to 5 cm.

### 2.2. Data Processing

Due to the high sensitivity and resolution of optical fiber monitoring technology, some noise signals may appear in the monitoring data. In order to eliminate the interference of noise signals, the appropriate noise reduction and smoothing methods are used to improve the signal-to-noise ratio of the monitoring data. The wavelet-improved threshold denoising method [23] can achieve high time resolution and low-frequency analysis in the high-frequency signal part, and high-frequency and low time resolution analysis in the low-frequency signal part. It has good adaptability and can be used for denoising processing of distributed optical fiber signals. It can effectively overcome the problems of continuity and deviation in hard thresholding and soft thresholding methods. In this study, we selected the wavelet type DB4 and set the threshold to 50%.

The formula for the wavelet transform is as follows. When $\Psi \left(t\right)\in L^{2}\left(R\right)$ satisfies the following formula conditions

(1) $$C_{\Psi }=\int_{R}\frac{{\left|\Psi \left(\omega \right)\right|}^{2}}{\left|\omega \right|}d\omega <\infty$$

The mother function Ψ(t) is called a mother wavelet. After scaling and translating the mother wavelet, the wavelet sequence can be obtained:

(2) $$\Psi_{a,b}\left(t\right)=\frac{1}{\sqrt{\left|a\right|}}\Psi \left(\frac{t-b}{a}\right)\;a,b\in R;a\neq 0$$

In Equation (2), *a* is the scaling factor, corresponding to the frequency and bandwidth of the change, and *b* is the translation factor, corresponding to the change in time.

This study uses the adjacent average method [24] based on the sliding filter principle for data smoothing processing. The adjacent averaging method sets a fixed-length sliding window, moves along the time series, performs arithmetic averaging on the signal data within the window, and then replaces the old data with the new smoothed data. By replacing them one by one, it is possible to effectively smooth the monitoring data curve.

The formula for the sliding adjacent average method is as follows:

(3) $$y\left[t\right]=\frac{1}{L}\sum_{k=0}^{L-1}x\left[t-k\right]$$

In Equation (3), *L* is the width of the sliding window. In this study, *L* is selected as 20.

### 2.3. Three-Dimensional Numerical Simulation Calculation Model and Parameters

**Figure 3:**
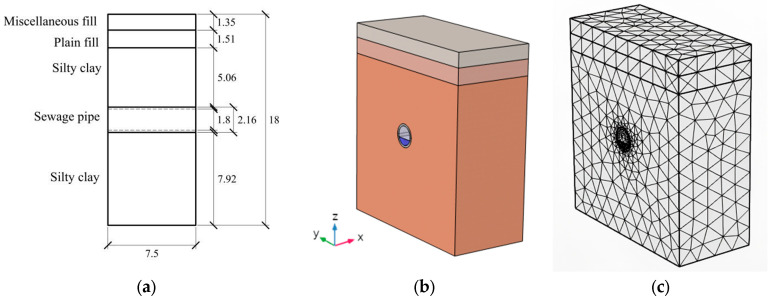
Underground sewage pipeline model: (**a**) cross-section of model (m); (**b**) numerical model; and (**c**) global meshing.

The numerical analysis model of the pipeline is shown in Figure 3. The numerical model is cuboid, with a miscellaneous fill soil layer, a plain fill soil layer, and a silty clay layer placed successively from top to bottom; the thicknesses are 1.35 m, 1.51 m, and 15.14 m, respectively. The sewage pipeline is a reinforced concrete pipeline with an outer diameter of 2.16 m and an inner diameter of 1.8 m. The center of the pipeline is 9 m away from the top and bottom surfaces of the model. The model has a height of 18 m, a width of 16 m, and a length of 7.5 m.

According to the operation of the pipeline, the sewage pipeline is not completely filled with water. The height of the water body in the model pipeline is set to 62 cm, accounting for 30.6% of the internal space of the pipeline. The physical field control network is used to divide the model grid so that the grid resolution of the area near the key pipeline is higher, and the grid resolution of the area with less influence on the edge soil can be lower. The numerical simulation is modeled using COMSOL Multiphysics 6.2. The overall model consists of 19,747 domain elements, 3542 boundary elements, and 428 edge elements. The underground sewage pipeline on Jianning Road is actually composed of multiple pipe sections spliced together. The pipe section connection structure includes various materials, such as steel rings, gaskets, rubber waterstops, etc., and the related parameters are complex. This simulation ignores the influence of various parameters at the pipe section connection interface on the temperature changes in the pipeline. The pipeline is modeled as a complete circular pipe, with a total of 2720 domain elements in the pipe model. According to the geotechnical investigation report and the relevant information of pipeline engineering, the model parameters are shown in Table 1.

The underground sewage pipeline is made of reinforced concrete material. The pipeline model parameters for this simulation are selected based on the literature and experience, as shown in Table 2. The thermodynamic parameters of water and air in the pipeline are selected by the COMSOL built-in empirical parameters.

## 3. Results

### 3.1. Pipeline Temperature Results

The strain temperature coefficient of the plastic-armored temperature optical fiber cable used in this experiment is 37 με/°C. By converting the strain information measured by the temperature optical fiber cable, the temperature change in the pipeline can be obtained. The underground sewage pipeline in the experimental section is composed of 20 sections of pipes with a length of 50 m. This study selects the temperature results of three sections of pipes (with a total length of 7.5 m) for analysis.

To conduct a more systematic and comprehensive study on temperature variations in sewage pipelines, this research also recorded the ambient temperature fluctuations on the monitoring day, as shown in Table 3.

**Figure 4:**
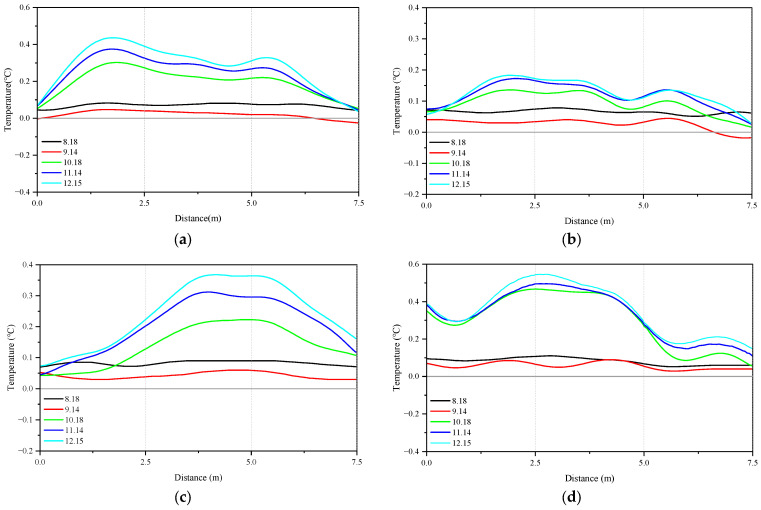
Variation in pipeline temperature: (**a**) right; (**b**) left; (**c**) top; and (**d**) bottom.

Figure 4 shows the temperature variation curves at four positions of the pipeline. The temperature changes in the pipeline monitored on August 18 and September 14 were relatively small; there were no significant fluctuations along the length of the pipeline. On 14 September, the temperature changes at four locations of the pipeline decreased compared to 18 August. After the sewage pipeline was officially opened to wastewater, the monitored temperature changes in the pipeline increased significantly from 18 October and gradually increased over time. From the temperature changes at four locations, it can be seen that due to the incomplete filling of the pipeline with sewage after water supply, the temperature changes on the right, left, and top plates are all smaller than those on the bottom plate. The temperature fluctuation amplitude of the bottom plate of the pipeline is larger than that at other positions. This is due to the presence of sewage at the bottom of the pipeline, which makes the bottom more sensitive to changes in pipeline temperature. The maximum temperature change in the pipeline is 0.55 °C, the minimum value is −0.03 °C, and the overall temperature rise is small.

### 3.2. Simulation Results

In the numerical simulation, the solid and fluid heat transfer are considered, the sewage in the pipeline is set as a laminar flow, and the conjugate heat transfer coupled with multiple physical fields is used. The model environment temperature is set to 15 °C, the sewage temperature inside the pipeline is set to 15.6 °C, the soil boundary is thermally insulated, and the laminar normal inflow velocity is set to 0.5 m/s. Based on laminar flow, the steady-state results are solved, and the solid and fluid heat transfer are incorporated to solve the transient results in conjugate heat transfer multiphysics fields. The actual on-site monitoring time interval is affected by weather and other factors; the average interval is 30 days, and there are five monitoring times. Considering the impact of sewage pipeline water flow, the numerical simulation selected 14 September 2021, as the starting time, with 10-day intervals to obtain the temperature change in the pipeline after water flow.

**Figure 5:**
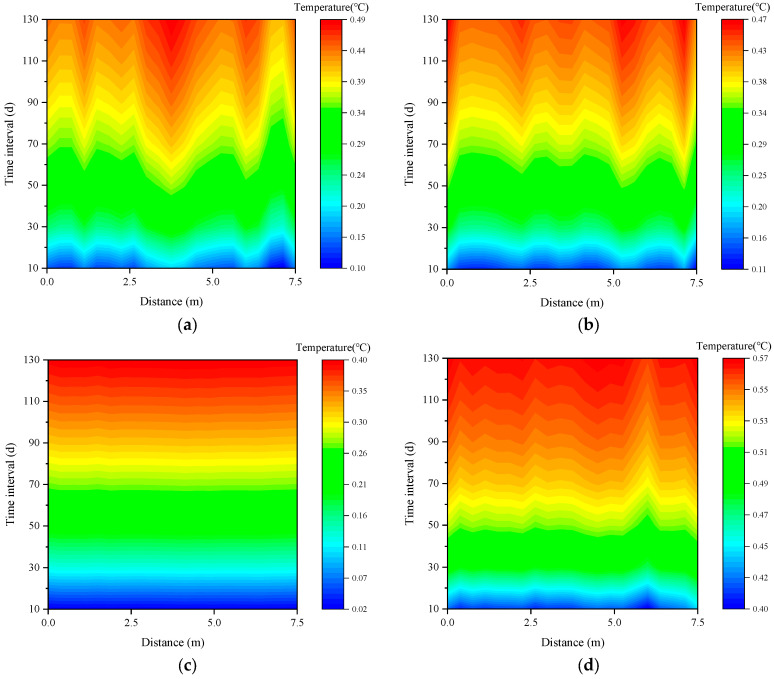
Numerical result of pipe temperature changes: (**a**) right; (**b**) left; (**c**) top; and (**d**) bottom.

Figure 5 shows the simulation results of temperature changes at four positions of the pipeline. After 130 days, the temperature changes at the four locations of the pipeline are 0.49 °C on the right side, 0.47 °C on the left side, 0.40 °C on the top plate, and 0.57 °C on the bottom plate. The temperature fluctuation of the pipeline is relatively small.

## 4. Discussion

### 4.1. Pipeline Temperature Change

**Figure 6:**
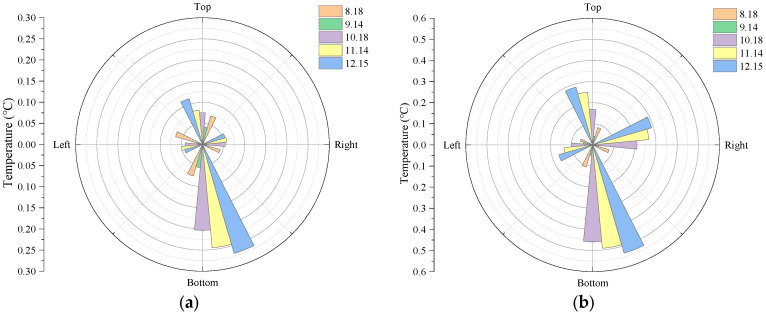
Pipeline temperature changes at different section positions: (**a**) section I and (**b**) section II.

The temperature changes at the different cross-sectional positions of the pipeline are shown in Figure 4. The temperature change in section I is shown in Figure 6a; the maximum value occurs on 15 December, with the bottom plate temperature of 0.26 °C. The temperature change in section II is shown in Figure 6b; the maximum value also occurs on 15 December, with the bottom plate temperature of 0.53 °C. For the different cross-sectional positions of the pipeline, due to the deep burial of the pipeline and the relatively constant temperature of the surrounding soil layer, the main driving factor for temperature changes in the pipeline is the sewage flowing inside the pipeline. The temperature changes at both cross-sectional positions are not significant.

**Figure 7:**
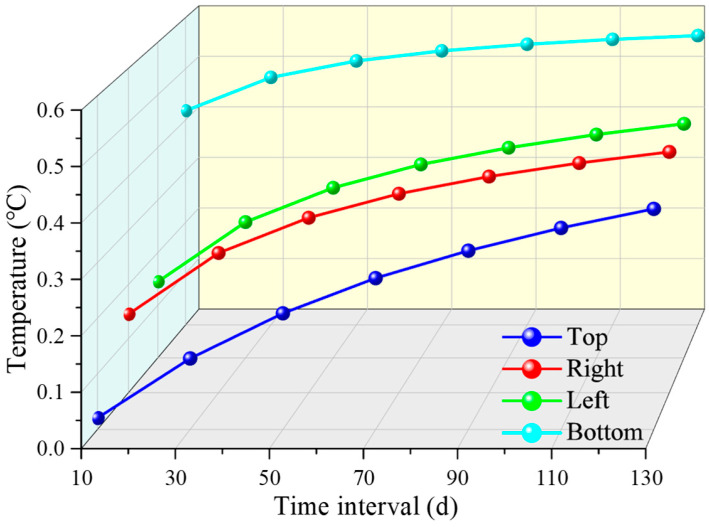
Average pipeline temperature changes at different positions.

The average temperature changes in the pipeline are obtained at different positions within a range of 7.5 m along the length direction. As shown in Figure 7, the average temperature change at the four positions of the pipeline is slowly rising during the simulation time, and the rising speed gradually slows down over time. Assuming that the water temperature inside the pipeline does not change, the temperature change at the bottom of the pipeline first reaches a stable state, followed by the left and right sides, and finally the top.

### 4.2. Comparison and Verification

**Figure 8:**
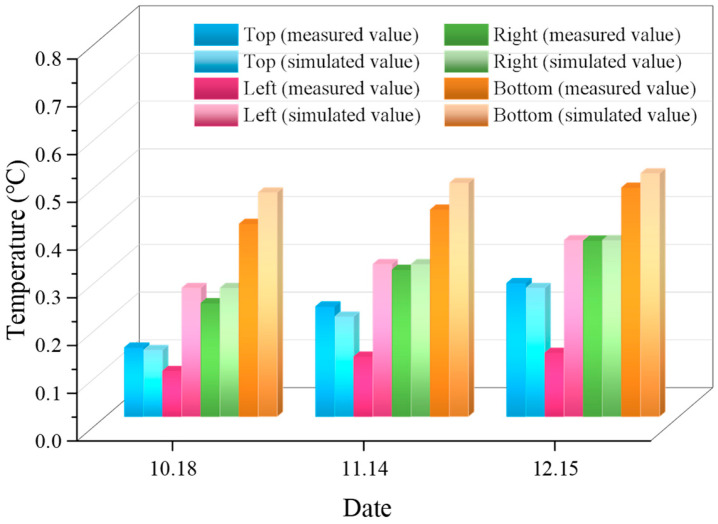
Comparison of simulated and measured results of pipe temperature changes.

At section I of the pipeline, 14 September is the reference date; the measured temperature changes in the pipeline on 18 October, 14 November, and 15 December are compared with the numerical simulation results. As shown in Figure 8, the numerical simulation results on the right side, top, and bottom of the pipeline are in good agreement with the measured results, with a maximum error of 16.34%. The measured results on the right, left, and bottom sides of the pipeline are slightly lower than the simulation results; the measured results on the top are higher than the simulation results. The trend of the numerical simulation results is consistent with the measured results, which indicates that the results of this numerical simulation are reasonable.

### 4.3. Conclusions

Temperature, as a critical factor influencing pipeline structural durability, serves as a key indicator for assessing pipeline health status. This study applies OFDR distributed fiber sensing technology to temperature monitoring of urban underground sewage pipelines, filling a technological gap in this field and providing innovative solutions for safe operation and maintenance. Through long-distance, high-precision temperature monitoring, we achieve accurate detection of subtle temperature variations in pipelines. In practical engineering applications, this technology effectively addresses the limitations of traditional detection methods in long-term, large-scale monitoring, avoiding the constraints of manual inspections and the risk of instrument damage. Combined with numerical simulation analysis, it enables more precise prediction of spatiotemporal temperature patterns, offering scientific support for pipeline design optimization, maintenance strategy formulation, and early fault warning. This has significant practical implications for ensuring the stable operation of urban underground pipeline networks, a critical lifeline infrastructure. The following conclusions can be obtained:

(1) The temperature inside the sewage pipeline continues to rise, and the rate of increase is relatively slow. According to the monitored temperature changes in the pipeline, the maximum temperature change in the pipeline is 0.55 °C, the overall temperature rise is relatively low, and the impact on the pipeline is relatively small. Due to the deep burial of the pipeline, the temperature of the surrounding soil layer is relatively constant, and the external environmental temperature has no effect on the temperature changes in the pipeline. The main driving factor for the temperature changes inside the pipeline is sewage.
(2) The numerical simulation results show that the results on the right, top, and bottom of the pipeline are in good agreement with the measured results; the maximum error is 16.34%. The measured results on the right, left, and bottom sides of the pipeline are slightly lower than the simulation results; the measured results on the top are higher than the simulation results. The numerical simulation results are consistent with the trend of the measured results. The results of on-site experiments and numerical simulations indicate that the temperature of sewage pipelines can be monitored by using OFDR distributed optical fiber sensing technology.

### 4.4. Limitations

While this study has yielded valuable insights through a combination of field experiments and numerical simulations, certain limitations remain. The experimental research focused on monitoring a 50 m pipeline section, with temperature data analyzed only for partial segments, which may not fully capture the temperature variation characteristics of the entire sewage network. Additionally, although noise reduction was applied to monitoring data during the experiment, the complex underground environment may still contain residual interference factors that could compromise data accuracy.

Numerical studies inherently involve assumptions and constraints. While incorporating multi-layered geological conditions with varying thermal and mechanical parameters, these models assume homogeneous properties across soil layers. However, natural soils exhibit significant spatial variations in thermal conductivity, moisture content, density, and other critical attributes. This oversimplified homogeneous treatment may compromise thermal transfer predictions, thereby affecting the accuracy of pipeline temperature distribution simulations. Furthermore, the model neglects the temperature effects of material interfaces (e.g., steel rings, gaskets, and rubber sealing strips) at pipe joints, reducing pipelines to perfect circular tubes that differ from actual spliced structures, potentially causing simulation discrepancies. Additionally, numerical modeling employs idealized conditions for wastewater flow dynamics and temperature changes within pipelines, whereas real-world engineering experiences dynamic variations in wastewater composition, flow velocity, and temperature, which may further compromise simulation accuracy.

## Figures and Tables

**Table 1:** Parameters of soil.

Soil Layer	Soil Thickness (m)	Density (kg/m^3^)	Poisson’s Ratio	Soil Water Content (%)	Young’s Modulus (MPa)	Specific Heat Capacity (J·kg^−1^·K^−1^.)	Thermal Conductivity (W·m^−1^·K^−1^.)
Miscellaneous fill	1.35	1734	0.28	23.2	10.00	996	0.90
Plain fill	1.51	1943	0.33	27.7	19.00	1264	1.15
Silty clay	15.14	1993	0.32	25.5	35.00	1580	1.54

**Table 2:** Parameters of sewage pipeline.

Pipeline Material	Density (kg/m^3^)	Thermal Conductivity (W·m^−1^·K^−1^)	Constant Pressure Heat Capacity (J·kg^−1^·K^−1^)	Inside Diameter (m)	Wall Thickness (mm)
Reinforced concrete	2040	1.56	880	1.80	180

**Table 3:** Monitor the daily temperature changes.

Date	Maximum Temperature (°C)	Minimum Temperature (°C)	Daily Average Temperature (°C)
8.18	31.0	24.0	27.5
9.14	29.0	25.0	27.0
10.18	28.0	21.0	24.5
11.14	20.0	6.0	13.0
12.15	15.0	6.0	10.5

## Data Availability

The data presented in this study are available upon reasonable request.
